# Experiential traces first: Does holding a location in visuospatial working memory affect the processing of space-associated words?

**DOI:** 10.3758/s13421-023-01512-5

**Published:** 2024-01-09

**Authors:** Oksana Tsaregorodtseva, Barbara Kaup

**Affiliations:** Department of Psychology, Language and Cognition Research Group, University of Tübingen, Schleichstr. 4, 72076 Tübingen, Germany

**Keywords:** Embodied language comprehension, Mental simulation, Visuospatial working memory, Attention

## Abstract

This study aimed to systematically examine whether actively maintaining a visual location in working memory can influence the processing of spatially related words. In five experiments, we asked participants to maintain either the location or the shape of a visually presented stimulus in working memory so that it could later be compared with a test stimulus concerning the relevant target features. In between, we presented participants with words that refer to objects typically encountered in the upper or lower vertical space (*roof* vs. *root*, respectively). The task participants performed as a response to these words differed between experiments. In Experiments 1–3, participants performed a lexical decision task, in Experiment 4 they performed a semantic task (deciding whether the word refers to an occupation), and in Experiment 5 they performed a spatial task (deciding whether the word refers to something in the upper or lower visual field.) Only in Experiment 5 did we observe an interaction between the position of the visual stimulus held in working memory (up vs. down) and the meaning of the spatial words (associated with up vs. down). Our results therefore suggest that actively maintaining a stimulus location in working memory does not automatically affect the processing of spatially related words, but does so if the relevant spatial dimension is made highly salient by the task. The results are thus in line with studies showing a strong context-dependency of embodiment effects and thus allow the conclusion that language processing proper is not operating on a sensorimotor representational format.

## Introduction

Numerous studies have provided evidence that the rehearsal mechanisms in visuospatial working memory involve attentional mechanisms (Awh et al., 1998; Smyth, 1996; Smyth & Scholey, 1994) and the programming of eye movements (Theeuwes et al., 2005; Theeuwes et al., 2009). Similarly, the processing of words referring to objects with a typical location (e.g., *roof*→ up vs. *root*→ down) has been found to trigger attentional shifts (Dudschig et al., 2012; Ostarek & Vigliocco, 2017) as well as the programming of eye-movements to the respective directions (Dudschig et al., 2013; Ostarek et al., 2018). These results suggest that the two processes may share mental resources. In the present study we combined these two lines or research, investigating whether actively maintaining a visual location in working memory affects the processing of spatially associated words. Before turning to the details of our study, we will outline the relevant research in both domains, visuospatial working memory and embodied language comprehension.

### Visuospatial working memory

Working memory can be defined as the ability to maintain and manipulate information that is currently inaccessible to perception. It has indeed been shown that the cognitive system can treat an object actively held in working memory as if it was available for perception. For instance, data suggest that attention and working memory are linked. In particular, the benefits of processing at an attended location (Posner et al., 1980) replicate in a working memory task (Awh et al., 1998; Awh & Jonides, 2001). Similarly, relocation of attention from a memorized location leads to a decline in memory performance for that location (Awh et al.,^1998^; Smyth & Scholey, 1994; Smyth, 1996).

In a seminal study, Awh et al. (1998) engaged participants in a dual-task paradigm. The first task was to memorize the location of a stimulus and perform a location recognition at the end of each trial. The second task was performed during the retention interval. Participants were presented with two types of letter-like probes either at the same location as the memorized stimulus (congruent trials) or at a different location (incongruent trials). The task was to identify which of the two letter-like probes was presented. It was hypothesized that participants’ performance during the retention interval would benefit from the memorized location in congruent trials. In contrast, in incongruent trials, the RTs were expected to be slower. The results confirmed this prediction by showing enhancement of processing at the memorized location. No such improvement was obtained when participants memorized an object shape instead of the location. In another experiment from the same study, Awh and colleagues showed that memory performance is reduced when attention needs to be shifted away from a memorized location. The authors interpreted the results in favor of the idea that attention is the mechanism that functionally supports spatial rehearsal in visuospatial working memory. In line with these findings, brain-imaging studies show that the same neural circuits in the frontal and parietal regions are activated when attention is endogenously directed to a cue and when the cue is stored in memory (Awh & Jonides, 2001; Corbetta et al., 2002). So, while attention is certainly occupied by relevant information that is present in the environment, it is also engaged when the information is stored in working memory.

Attention is not the only mechanism linked with working memory. It is well known that eye movements are directed to the relevant targets in space (Deubel & Schneider, 1996; Kowler et al., 1995). Likewise, phenomena such as “looking at nothing” (Ferreira et al., 2008) indicate that our eye gaze follows our memory processes: remembering something goes hand in hand with looking at the associated location. Memory can also recruit more complex eye-movement patterns. For instance, it has been shown that the oculomotor system is involved when a visual distractor that needs to be ignored appears during a saccade towards a target stimulus (Doyle & Walker, 2001; Godijn & Theeuwes, 2002; Sheliga et al., 1994). In particular, in the study of Doyle and Walker (2001), observers had to execute a saccade to a color singleton and ignore a distractor. The results showed that saccades deviated away from the abruptly appearing distractor. Similar results were obtained for objects held in working memory. In a study by Theeuwes et al. (2005), participants either memorized the exact location of a dot presented in different positions on the screen or just observed the dot. Then a saccade had to be made into the upper or lower target area. Saccades to the target area deviated away from the remembered location, but only if it was the location of the stimulus that participants had to memorize. Thus, the studies by Doyle and Walker (2001) and Theeuwes et al. (2005) showed similar result patterns. The difference was that, in one case, the saccade deviated from the visually accessible object, and in the other case, the saccade curved away from the remembered object. The curvature in both cases is attributed to the need to inhibit the programming of eye movements to a location that needs to be remembered or ignored. Traditionally, this mechanism has been considered a competitive activity in the superior colliculus, a low-level structure in the midbrain that works as a motor map for saccade generation to visual stimuli as potential targets (Theeuwes et al., 2005; see Theeuwes et al., 2009, for a review).

Taken together, the reported empirical results indicate a link between working memory, attention, and eye movements. In addition to pointing towards this link, the reported empirical results are also in line with the two most influential conceptualizations of working memory: Baddeley and colleagues’ multi-component model (Baddeley, 1998; Baddeley & Hitch, 1974) and the model of Cowan (Cowan, 1988; Cowan, 1993; Cowan, 2000). According to the model initially proposed by Baddeley and Hitch (1974), working memory consists of two modality-specific sub-systems for storing verbal or visuospatial information and a central executive system that controls these sub-systems. Later, an episodic buffer was added as a component explaining the interaction between stimuli of different modalities (Baddeley, 2000). Baddeley argued that eye-movement programming is a rehearsal mechanism responsible for storing and updating objects in visuospatial memory. For verbal information, an equivalent role is played by the phonological loop. Cowan (Cowan, 1988, 1993, 2000) in contrast does not posit separate systems for verbal and visuospatial information in his model. According to his model, working memory information is rehearsed by means of an attention-based mechanism that selects the relevant subset of information from long-term memory. Thus, everything in the focus of attention is effectively in working memory. Thus, across both views, objects are maintained in working memory by means of attention and eye movements, which are the very mechanisms required for responding to objects available to perception.

### Embodied language comprehension

Embodied theories of human cognition (Barsalou, 1999; Barsalou, 2008;Binder & Desai, 2011 ; Pulvermüller, 2013) offer an alternative viewpoint to the symbolic and amodal perspective of cognition (e.g., Fodor, 1975). According to the symbolic view, language comprehension is a symbolic process, separate from other modalities in the human brain. It suggests that language activates specific language areas such as Broca and Wernicke, emphasizing their role in understanding language. In contrast, the embodied view proposes that language comprehension is a distributed process involving various brain areas responsible for action, perception, and emotion (Binder & Desai, 2011; Pulvermüller, 2013). In this framework, language is inherently connected to action, perception, and emotion, requiring the integration of multimodal experiential traces for comprehension. It has been suggested that humans associate words with the experience of the corresponding objects, situations, and events during language acquisition. These experiential traces, in turn, contribute to their understanding of language when they later encounter the words without their referents being present. For example, when a word like “sun” or “cloud” is being learned, children often look up or watch someone pointing up, while for words like “grass,” they lower their heads or see someone pointing down. From an embodied cognition perspective, these experiential traces get re-activated when hearing words like “sun” or “grass,” in a situation in which these objects are not present, enriching the comprehension process.

Following this logic, Dudschig et al. (2012) conducted an experiment in which participants were presented with spatially associated words such as “sun” or “grass” and afterwards identified targets located either in the upper or lower part of the screen. It was shown that the processing of the spatially associated words facilitated the subsequent identification of targets located in positions consistent with the positions of words’ referents. Specifically, a word like “sun” enhanced target identification at the top of the screen whereas a word like “grass” enhanced target identification at the bottom of the screen, suggesting that these words triggered attention shifts to the corresponding location. Similar findings were observed in a study by Tsaregorodtseva and Miklashevsky (2015), in which participants performed a target discrimination task. Similarly, Ostarek and Vigliocco (2017) demonstrated facilitation effects when targets were pictures associated with briefly presented object words. However, in contrast to the facilitation effect described so far, Estes et al. (2008) observed an inhibition effect when participants discriminated targets (X or O) following the presentation of spatially associated words. The authors attributed this effect to the perceptual simulation of the word’s referent in a corresponding location, which presumably impeded target discrimination at that location. This inconsistency in the direction of the effects has been extensively discussed in theTsaregorodtseva & Miklashevsky, 2015 literature (Dudschig et al., 2012; Estes & Barsalou, 2018; Ostarek & Vigliocco, 2017). Notably, Estes and Barsalou (2018) argued that the direction of the effect depends on various factors, including the task, the temporal interval between the word and the target, and the characteristics of the orthographic system (see Estes & Barsalou, 2018). Despite the ongoing debate surrounding the direction of the effect, for our purposes in this study it is crucial to highlight that spatially associated words exhibit a similar effect to that of symbolic visual cues in directing attention (Langton et al., 2000; Posner et al., 1980). In essence, these words possess the ability to guide attention in a manner comparable to purely symbolic visual cues.

Research has also demonstrated that spatially associated words can engage the oculomotor system. For instance, Dudschig et al. (2013) found that saccades are launched faster in an upward direction after reading words like “sun” or “cloud,” as compared to a downward direction whereas the opposite holds for words like “grass” or “root.” Additionally, Ostarek et al. (2018) explored saccade trajectories instead of saccade launches and discovered that the processing of spatially associated words influenced vertical saccade trajectories towards directions congruent with the spatial associations of the words. Furthermore, studies involving participants performing hand movements (Dudschig et al., 2013) or using grip force sensors (Miklashevsky, 2022) while reading spatially associated words indicated the involvement of the motor system. This suggests that these words can effectively engage the motor system, simulating the presence of actual objects in space.

Indeed, embodiment effects extend beyond spatially related words, encompassing various meaning aspects in language processing. For instance, Bub et al. (2008) presented evidence that language can activate specific motor plans. They demonstrated that words referring to manipulable objects evoke corresponding grasping actions similar to when participants view images of those objects. Similarly, Glenberg and Kaschak (2002) observed compatibility effects between arm movements and sentences that implied actions involving arm movements, such as *He closed the drawer* versus *He opened the drawer*, indicating a link between language and motor representations (for a recent meta-analysis on this effect, see Winter et al., 2022). Furthermore, language has also been shown to activate modality-specific visual representations of object shape (Huettig & Altmann, 2004; Huettig & Altmann, 2007; Kaup et al., 2006; Ostarek & Huettig, 2017; Zwaan et al., 2002), object orientation (Stanfield & Zwaan, 2001), or even object color (Connell, 2007; Mannaert et al., 2017; Tsaregorodtseva et al., 2023; Zwaan & Pecher, 2012). All these studies endorse the view that language engages the sensorimotor system, suggesting a close relationship between language comprehension and cognitive processes related to perception and action.

### The current research

In the current study, we aimed at investigating whether maintaining a location in visuospatial working memory would affect the processing of spatially associated words, as suggested by the literature review showing that visual-spatial working memory and processing of spatially associated words share cognitive resources. To examine this question, we adopted a paradigm commonly utilized in working memory research (Awh et al., 1998; Belopolsky & Theeuwes, 2009; Theeuwes et al., 2005). Participants were instructed to memorize a visual location, as in previous studies. However, rather than exploring the influence of the remembered location on the processing of a stimulus at a certain target location, we explored its impact on the processing of spatially associated words.

In addition to investigating the relationship between visuospatial working memory and language processing, there is another aim of the present study. An important debate in the embodied language processing literature concerns the question what role sensorimotor meaning representations play for language processing. Critics of the embodied view of language processing argue that most of the reported embodied effects are only a by-product of language processing (Leshinskaya & Caramazza, 2016), resulting from the spreading of activation across brain regions (see also Mahon & Caramazza, 2008). According to this view, language processing proper does not operate on a sensorimotor (modal) format, and representations in this format are therefore not functionally relevant for language processing. Accordingly, many authors in the embodied language processing community have pointed out that evidence concerning the causality of embodied meaning representations for language processing is of central importance (e.g., Ostarek & Huettig, 2019; see also Kaup et al., 2016). In this respect, one shortfall of the studies available today in that literature is that a vast majority of these studies looks at influences in one direction, namely from language processing (involving for instance a word or sentence reading task) to processing in a non-linguistic domain (involving for instance sensorimotor processes such as pressing an upper key, pulling a lever or classifying a certain color patch). However, if non-linguistic or sensorimotor processes are indeed functionally relevant for linguistic processing then similar influences in the opposite direction should exist. In other words, there should be clear evidence for a direct influence in the other direction, such that linguistic processing proper is influenced by some previous non-linguistic or modal process. Only a few studies actually looked at influences in this “other” direction, and the results are mixed. Pretty strong evidence was provided by a study by Glenberg et al. (2008). Participants were initially engaged in a task involving the movement of beans, either away from or towards their bodies, for approximately 20 min. Following this, they were required to make sensibility judgments regarding sentences describing the transfer of objects in three possible directions: towards the reader, away from the reader, or no transfer at all. The findings revealed that participants exhibited slower performance when the sentences described a transfer direction that aligned with their prior bean manipulation practice. From these observations, the authors concluded that the preactivated motor system had an impact on language comprehension. However, a series of studies involving an anagram solving task provided weaker evidence. A study by Berndt et al. (2020) for instance found that a compatible modal cue (e.g., a visually presented color patch) facilitated an anagram solving task involving color-related words (e.g., the word “cucumber” written as an anagram *cbemcuru* after seeing a green color patch). In contrast, anagrams for spatially related words were solved faster after a modal cue (appearing at the top of the bottom of the screen) only under certain conditions, namely when an additional supportive scene was placed in the background (Berndt et al., 2018). In other words, the influence of non-linguistic processes on linguistic processes was less clear than predicted by strong versions of the embodied cognition view. Thus, we consider it highly relevant for the embodied view of language comprehension to gain more evidence regarding influences of a non-linguistic processing domain to linguistic processing proper. In the present experiments, we look at exactly these types of influences, namely from memorizing a stimulus location in visuospatial working memory (modal cue/non-linguistic processing) to processing of spatially related words (linguistic processing). Our experiments therefore potentially provide much needed evidence for the embodied language processing view.

Another important issue in the debate about the role of embodied meaning representations for language processing concerns the context-dependency of embodied effects (e.g., Lebois et al., 2015; Huettig et al., 2020; see Yee & Thompson-Schill, 2016, for an overview). For instance, Tsaregorodtseva and Miklashevsky (2015) investigated the effect of the spatially associated words on the processing of a subsequently presented visual target, which was observed only in a target-discrimination but not in a target-identification task. Also, Pecher et al. (2010) demonstrated task-dependency when presenting “sky” versus “ocean” words at the top versus bottom of the screen to investigate compatibility effects between presentation location and word meaning, whereby a similar study by Šetić and Domijan (2007) had observed such compatibility effects with a slightly different task. Similarly, strong task-dependency could also be demonstrated in a recent study by Tsaregorodtseva et al. (2023) targeting compatibility effects between the processing of linguistic stimuli referring to objects with a typical color (*cucumber* vs. *tomato*) and pressing a colored response button (e.g., red vs. green). Stable compatibility effects were only observed with word stimuli, whereas for sentence materials compatibility effects were only observed when there were no fillers in the materials and the relevant color categories were thus highly salient (see also Dudschig & Kaup, 2017). Obviously, context-dependency is another issue that might cast doubt when it comes to strong versions of the embodied language processing account, claiming that modal representations are functionally relevant for language processing. We address this issue by manipulating between experiments the linguistic task that participants perform with the spatially associated words after memorizing a target stimulus in visuospatial memory.

For our experiments, we recruited participants either by email to students at the University of Tübingen (Experiments 1a and 2–5) or by using a combination of this collection method and using the Prolific platform (Experiment 1b). When we collected data via email, we asked participants to choose from either a course credit or a €5 reimbursement. When the data was collected through Prolific, participants were paid £4.67. The survey link instructions requested the workers to be native German speakers. At the beginning of the task, participants gave informed consent and were further inquired on their native language. Workers who had already participated in one of the tasks were excluded from further participation. The experiments were implemented by means of jsPsych (De Leeuw & Motz, 2016) and participants ran them on their computer or laptop using a common web browser. Participants typed their responses using their standard keyboard.

## Experiment 1

In this experiment we ran two sub-experiments with different tasks to see whether the memorized location would influence the processing of spatially related words. We did this following the study of Awh et al. (1998), who investigated the effect of memorized location on recognizing the actually presented location. Specifically, Awh et al. (1998) ran two consequent experiments, in one of which participants memorized the location of a target object, whereas in the other participants memorized the shape of the target object. Awh et al. (1998) contrasted the results of these two tasks to demonstrate that the effect of the memorized location was actually the result of keeping the location in the memory, not the result of cognitive load. Similarly, in Experiment 1a of the current study, the participants’ task was to memorize the exact location of a dot that could appear in the upper or lower parts of the screen. In contrast, in Experiment 1b, the task was to memorize the shape of the figure instead of the exact location. We assumed that these two different context tasks would have a different effect on our primary task that appeared during the retention interval and involved the decision about space-associated words. More specifically, we predicted that an effect of maintaining a stimulus in working memory would only affect the subsequent processing of spatially associated words in case participants had to keep the location in memory not in case they had to keep the shape in memory. We report Experiment 1a fist, and then turn to Experiment 1b.

### Method of experiment 1a

#### Participants

We collected data of 112 volunteers^1^ (32 men; *M*_age_ = 24.50; *SD* = 8.06 years), of whom 109 declared German as their mother tongue (32 men; *M*_age_ = 24.40; *SD* = 8.06 years). These served as the final sample and their data was used for further analysis.

#### Materials

We used 80 German words referring to entities typically associated with a position in the upper or lower visual field (Dudschig et al., 2013; Lachmair et al., 2011). The stimuli were rated in previous studies according to the location of their referents in the real world, showing clear differences in rated position: *t*(78) = -41.94, *p* <.001. The words in the two categories did not differ significantly with regard to frequency: *t*(78) = -0.17, *p* = .864, or length: *t*(78) = 0.39, *p* = .701).

Additionally, we used 40 fillers which were words that did not exhibit an association with the upper or lower location of their referents according to the rating study reported in Lachmair et al. (2011). We also created 120 pseudowords using a pseudoword generator Wuggy (http://crr.ugent.be/programs-data/wuggy).

#### Procedure and design

**Fig. 1:**
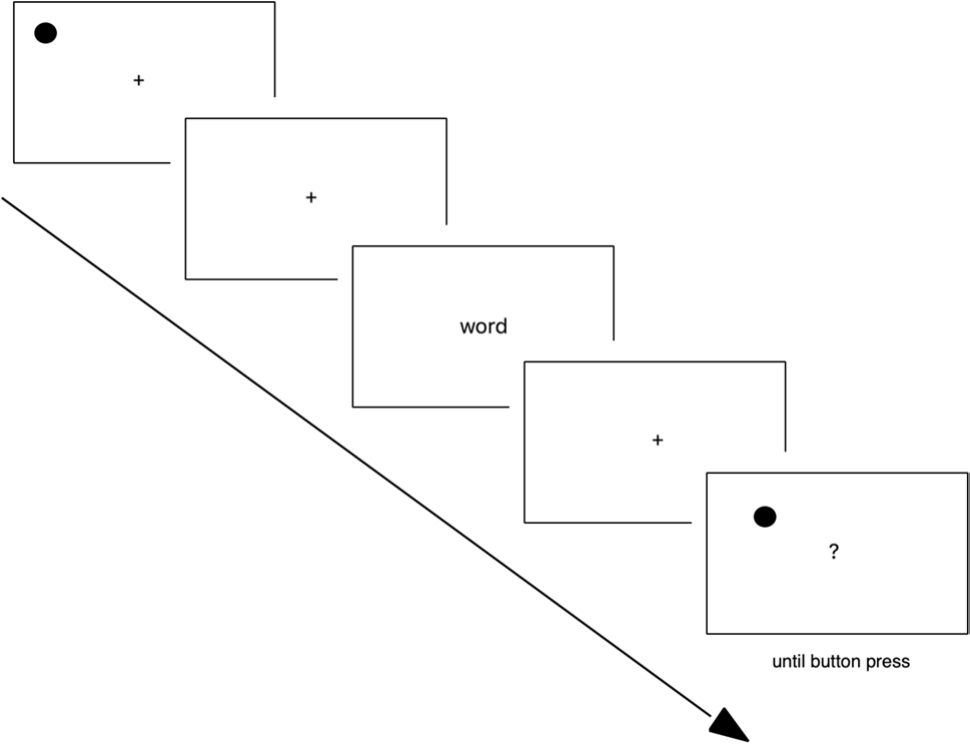
Schematic representation of trial procedure (not to scale)

Participants fixated on a centrally presented fixation cross for 500 ms. Then a dot appeared for 500 ms. The dot, 1.3 cm in diameter, appeared in one of the four 3.8 cm × 3.3 cm lateral cells (left/right top/bottom) of the screen divided into 3 × 3 grids. Grids were centered at x = ±3.5cm, y = ±3 cm from the central cross. The dots and cells had the same size on different participants’ screens by means of the *resize* plugin of jsPsych. The dot could appear in one of the 120 locations within the cells. Participants had to remember the exact location of the dot. Then a centrally presented cross appeared again and stayed on the screen for a randomly selected interval of 1,000, 1,500, or 2,000 ms. Afterwards, a word or pseudoword was presented for 1,500 ms. Participants performed a lexical-decision task using a keyboard as input device, pressing the keys “S” or “K.” After a retention interval of 1,000, 1,500, or 2,000 ms, also randomly determined, the recognition task followed. A dot was presented either at the same position as the earlier dot or at a slightly different position (in the same vertical half of the screen). The difference between the initial and second dots did not exceed 1.5 cm from edge to edge. Participants pressed again the “S” or “K” key on the keyboard to give their response (same/ different^2^). We gave error feedback of 5,000 ms if participants did not give the correct answer, both after the lexical-decision task and the dot-recognition task. The response buttons were counterbalanced for both tasks. A schematic representation of the trial is shown in Fig. 1. A practice session was administered prior to the experiment proper and contained ten trials. The practice session was repeated if participants did not give at least 80% of the correct answers. Trial order was randomized. The experiment took approximately 45–50 min.

We would like to point out that our paradigm was adapted from previous studies investigating visuospatial working memory (Awh et al., 1998; Belopolsky & Theeuwes, 2009; Theeuwes et al., 2009). In particular, as before, we placed the dots in the four lateral cells instead of directly above or below the central cross to prevent unintentional verbal coding (to prevent them from directly coding locations as “up” or “down”). Also, we made the difference between the first and second dots relatively small to engage active working memory maintenance.

The design of the experiment was a 2 (dot position: top vs. bottom) × 2 (referent position: up vs. down) design with both factors being manipulated within participants and within items. Response time and accuracy rates in the lexical-decision task served as dependent variables.

### Results and discussion of experiment 1a

#### Data cleaning

The first step before analysis was to identify the accuracy thresholds for the two tasks (dot-recognition and lexical decision). The mean accuracy for the dot-recognition task was 84%. We visually inspected participants’ performance using a scatterplot and a boxplot. Three participants clearly deviated from the rest, with their accuracy rates being below 60%. The data of those participants were considered outliers and were discarded from the analysis. The same procedure was applied for participants’ accuracy in the lexical-decision task. Overall, participants were better at performing this task (94%), and, accordingly, we applied a higher threshold in this task. We deleted the data of seven participants who did not reach a threshold of at least 90%. We ended up with a final sample of 99 participants (28 men; *M*_age_ = 24.12; *SD* = 7.63 years). The mean accuracy for these participants was 84% for the memory task and 95% for the lexical-decision task.

For analyzing the lexical-decision responses, we only took trials into account in which participants memorized the dot correctly, ensuring that the lexical-decision was indeed performed while holding the location of the dot in memory. This procedure resulted in 14% of data loss. Furthermore, we only took into account correct responses in this task, resulting in a further data loss of 3%. Based on visual inspection of the data plot, responses faster than 200 ms and slower than 1,100 ms were considered absolute outliers and were omitted (2.7% of the data). We then converted the RTs (RTs) to z-scores per condition and participant for relative outlier elimination. We eliminated all trials for which the absolute value of this z-score exceeded -2/+2 (1.9 % of the relevant data).^3^ We used this outlier elimination procedure in all of our experiments. The means of the final set of RTs are shown in Table 1.

**Table 1:** Means and standard deviations per condition in Experiments 1–2

	Experiment 1a		Experiment 1b		Experiment 2	
Referent position	Dot position		Dot position		Dot position	
	Top	Bottom	Top	Bottom	Top	Bottom
Up	684(95)	668(89)	681(83)	676(87)	715(107)	711(97)
Down	686(92)	682(92)	684(84)	681(83)	725(107)	722(103)

#### Response-time analysis

We analyzed the results by means of a linear mixed-effects model (LMEM) using the free statistic software R (Version 4.0.3) and the R-package lme4 (Bates et al., 2014). We implemented contrast coding for categorical variables.

When building a suitable model for our data, we first selected the random effect structure. To do that, we first built the models including fixed effects for “referent position” and “dot position,” their interaction, and different combinations of random effect structures. As models with complex random effects structures led to singular fit or convergence problems we ended up with a model with only random intercepts for participants and items. We then constructed the base model for further analysis. Our base model consisted of fixed main effects for “referent position” and “dot position” and random intercepts for participants and items. We then compared the base model to a reduced one to examine the main effects. A likelihood-ratio test showed that the base model was better than the reduced model without the factor “dot position” (χ2(1) = 9.8, *p =* .002, *d* = -0.08) but not better than the reduced model without the factor “referent position” (χ2(1) = 0.56, *p =* .455, *d* = 0.18), indicating a main effect of “dot position” and no main effect of “referent position.” To test our hypothesis, we also compared the base model with the model including an interaction. The comparison showed that the model with interaction did not explain the data significantly better than the base model (χ2(1) = 1.68, *p =.*195, *d* = 0.03). However, planned comparisons showed that there was a significant difference between top and bottom positions (684 ms vs. 668 ms, see Table 1) when participants made decisions about “up” words: (χ2(1) = 10.36, *p =* .001, *d* = -0.12). At the same time, when participants made decisions about “down” words, such a difference was not obtained: (χ2(1) = 1.48, *p =* .223, *d* = -0.05).

One could argue that data loss due to omitting all the trials in which participants had not responded correctly in the recognition task was too high. To make sure that we did not miss any effects due to this planned procedure of ours, we also analyzed the data, not excluding incorrect trials for the memory task. The results remained the same: The interaction was not obtained: (χ2(1) = 1.71, *p =.*191, *d* = 0.03) but we saw a simple main effect of dot position when participants made decisions about “up” words: (χ2(1) = 11, *p =* .001, *d* = -0.12), with means for the top and bottom position being 683 ms versus 670, respectively. As in the previous analysis, the same effect for “down” words was not observed: (χ2(1) = 2.05, *p =* .152, *d* = -0.05). The means for top and bottom positions were 685 ms versus 681 ms, respectively).

#### Accuracy analysis

The accuracy analysis showed no benefit of either condition (all *p*s≥ 1), indicating no noticeable speed-accuracy trade-off. The percentage of correct responses per condition is shown in Table 2.

**Table 2:** The percentage of correct responses in Experiments 1–2

	Experiment 1a		Experiment 1b		Experiment 2	
Referent position	Dot position		Dot position		Dot position	
	Top	Bottom	Top	Bottom	Top	Bottom
Up	95%	97%	96%	97%	95%	95%
Down	96%	96%	96%	96%	95%	95%

### Discussion

Experiment 1a did not show the expected interaction between the referent position and the dot position. It seems that holding a location in spatial working memory does not influence lexical decisions about words with spatial associations. Post hoc analyses, however, showed a main effect of dot position when participants decided about “up” words, which was in line with our prediction. It is worth noting that in previous studies examining words with spatial associations, also only the “up” words resulted in an effect (Dudschig et al., 2013; Janyan et al., 2015; Tsaregorodtseva & Miklashevsky, 2015). Thus, although the predicted interaction was not observed, the present results did replicate this asymmetry of the effect with a different paradigm. We ran Experiment 1b to see whether a feature other than location actively stored in memory would similarly influence the decision about words with spatial associations.

### Method of experiment 1b

#### Participants

The experiment involved 130 volunteers who did not participate in Experiment 1a (37 men; *M*_age_ = 24.14; *SD* = 4.95 years). Data from 125 participants who reported German as their first language were used for further analysis (32 men; *M*_age_ = 23.92; *SD* = 4.17 years).

#### Materials

The word material was the same as in Experiment 1a. For the shape-memory task, we used figures as letter-like stimuli of various shapes (e.g., ǂ or Ŧ).

#### Procedure and design

The procedure and design repeated the procedure and design of Experiment 1a with one exception. Instead of memorizing the location, we asked the participants to remember the exact shape of the stimuli.

### Results and discussion of experiment 1b

#### Data-cleaning procedure

The mean accuracy for the memory and lexical-decision task was comparable with the results of Experiment 1a (80% vs. 95%, respectively). In Experiment 1b, we followed the same data-cleansing procedure as in Experiment 1. First, we removed the data of four participants who completed the memory task below the level we set in Experiment 1a (i.e., below 60% of accuracy). The mean accuracy for the rest of the participants’ memory task was 81%. We also removed data from ten participants who did not pass the 90% accuracy threshold on the lexical-decision task. The mean accuracy of the remaining participants was 96%. All procedures resulted in a final sample of 111 participants (29 men; *M*_age_ = 24.06; *SD* = 4.19 years).

For the analyses of the lexical decision RTs, we selected only the trials in which participants correctly responded in both tasks, resulting in 18.4% and 2.76% data loss for the memory and lexical decision tasks, respectively. We considered responses faster than 200 ms and slower than 1,100 ms as absolute outliers and removed them (2.14% of the data). We followed the same procedure to eliminate relative outliers based on z-scores per condition and participant as before. We excluded all trials for which the absolute value of this z-score exceeded -2/+2 (1.99% of corresponding data). The means of the final set of RTs are shown in Table 1.

#### Response-time analysis

The base model contained fixed main effects for the referent position and dot position and random intercepts for participants and items. Other models with more complex structures of random effects did not converge. We followed the same procedure of analysis as in Experiment 1a. The analysis showed no main effect of “referent position” (χ2(1) = 0.41, *p =* .521, *d* = 0.14), and only a marginally significant main effect of “dot position” (χ2(1) = 3.09, *p =* .079, *d* =-0.04), reflecting faster lexical decisions when maintaining the “bottom” shape in memory than when maintain a “top” shape. Also, the model with interaction did not describe the data better than the model without interaction (χ2(1) = 0.09, *p =* .754, *d* = 0.01). Planned comparisons showed no effect of “dot position” in either the “up” words condition: (*χ*2(1) = 0.96, *p =* .326, *d* = -0.05), or the “down” words condition (χ2(1) = 2.06, *p =* .151, *d* = -0.04).

As in Experiment 1a, we re-analyzed the data by included trials in which participants responded incorrectly in the memory task. Similar to Experiment 1a, the interaction was not significant: (*χ*2(1) = 0.22, *p =.*639, *d* = 0.03) and neither were the simple effects for “up” and “down” words (all *p*s >.05).

#### Accuracy analysis

The accuracy analysis revealed no advantage of either condition (all *ps >* .1). The percentage of correct answers per condition is shown in Table 2.

### Discussion

Experiment 1b did not reveal an interaction between dot position and referent position as in Experiment 1a, but this time this was expected as the task involved shape memory instead of location memory. In contrast to Experiment 1a, however, there now also was no simple main effect for the “up” words condition in Experiment 1b, as expected. Of course, in Experiment 1a the respective difference was only a post-hoc effect which should be interpreted with caution considering the non-significant interaction. However, a similar pattern has been reported before in the literature involving other paradigms, and we do want to make sure that we are not missing a potentially important effect in the current paradigm. We therefore decided to run Experiment 1a again (location memory) but this time involving the slightly more visually complex stimuli from Experiment 1b. We considered it possible that these more complex stimuli would lead to a stronger engagement of visual-spatial working memory and thus potentially to a stronger influence of location memory on processing spatially associated words.

## Experiment 2

The goal of Experiment 2 was to replicate the results of Experiment 1a, but this time using more complex visual stimuli, which were the exact same stimuli as in Experiment 1b but with asking participants to memorize the location of the figures. Thus, compared to Experiment 1a, we changed the stimuli whose location was to be memorized (dots in Experiment 1a vs. letter-like objects in Experiment 2). Compared to Experiment 1b, we changed the context task (shape-memory task in Experiment 1b, location-memory task in Experiment 2). We were interested in finding out whether the same pattern of results would emerge as in Experiment 1a.

### Method

#### Participants

We recruited 134 participants (36 men; *M*_age_ = 25.58; *SD* = 9.04 years). Data cleaning resulted in the data set of 132 participants (35 men; *M*_age_ = 25.51; *SD* = 9.05 years).

#### Materials

The linguistic stimuli were the same as in Experiment 1. The visual stimuli employed in the context task were the same as in Experiment 1b.

#### Procedure and design

The procedure and design were identical to Experiment 1b except for the task. In Experiment 2, we asked participants to remember the exact location of the shapes. Thus, the presented material repeated Experiment 1b, but the task was the same as in Experiment 1a.

### Results and discussion

#### Data-cleaning procedure

The mean accuracy was 81% versus 94% for the memory and lexical decision task, respectively. Fifteen participants were excluded due to poor performance on the memory task (below 60%). Mean accuracy for the rest of the participants was 84%. We also omitted the data of 13 participants who did not pass the 90% threshold for the lexical-decision task. The mean accuracy for the rest of the participants was 95%. This procedure resulted in a final set of 106 participants (26 men; *M*_age_ = 25.15; *SD* = 8.63 years).

In the RT analysis, we again looked only at trials which led to correct responses in both tasks, resulting in 15.17% and 3.65% data loss for the memory and the lexical-decision tasks, respectively. We then excluded absolute outliers (3.4%) and relative outliers (1.75%) as before. The means of the final set of RTs are shown Table 1.

#### Response-time analysis

We followed the same analysis procedure as in Experiment 1. The base model included fixed main effects for the referent position and dot position and random intercepts for participants and items. We compared the base model with reduced models, not including the main effect of referent position or of dot position. The likelihood ratio tests show neither a main effect of dot position (χ2(1) = 2.41, *p =*.121, *d* = -0.04) nor a main effect of referent position (χ2(1) = 1.72, *p =*.19, *d* = 0.31). Also, the interaction of the two factors was not significant (χ2(1) = 0.17, *p =* .684, *d* = 0.01). Planned comparisons did not reveal any significant results, neither for “up” words: (χ2(1) =1.87, *p =* .172, *d* = -0.05) nor for “down” words: (χ2(1) =0.61, *p =* .436, *d* = -0.03).

Following the course of analysis performed in Experiment 1, we also analyzed the data without excluding incorrect trials for the memory task. The interaction between the referent position and dot position was not obtained (χ2(1) = 2.6, *p =* .107, *d* = 0.04). However, the simple main effect of dot position was significant for the “up” words (χ2(1) =5.26, *p =* .022, *d* = -0.08), with the means for top and bottom positions being 719 and 709 ms, respectively. The same was not observed for the “down” words: (χ2(1) =0, *p =* .997, *d<*0.01). Here the means were 723 ms for both positions. The pattern of results in this analysis thus replicated the results obtained for Experiment 1a.

#### Accuracy analysis

There was no accuracy advantage in either of the conditions (all *ps >* .3). The percentage of correct answers per condition is shown in Table 2.

### Discussion

The results of this experiment were mixed. On the one hand, our main analysis did not show evidence in favor of the idea that an actively maintained location in working memory affects decisions about space-associated words. The simple main effect observed in Experiment 1a was not replicated in Experiment 2, where participants also memorized a location. On the other hand, in our second analysis considering more available data, the respective effect was significant. We did not obtain the interaction, but the simple main effect was now predicted and demonstrated the same direction and appeared in both experiments in which participants memorized the location of a stimulus (Experiment 1a and Experiment 2) instead of its shape (Experiment 1b). In order to be absolutely sure that we do not overlook a small but reliable effect, we decided to replicate the experiment again but this time with a much larger sample size. A power analysis, applying the *simr* package functions on the basis of the data of Experiment 1a, showed that 99 participants gives a power of 90% for the simple effect of dot position for “up” words and 200 participants a power of 99%. We decided to increase our sample size to at least 200 participants for the next experiment in order account for the fact that effect sizes are often overestimated when estimated on the basis of a significant effect in previous research (Brysbaert, 2019; Gelman & Carlin, 2014; Vasishth et al., 2018)

## Experiment 3

Experiment 3 was aimed at replicating the results of Experiment 1a. The experiment was preregistered (https://aspredicted.org/gf7mw.pdf).

### Method

#### Participants

Two hundred and eighty-eight volunteers (70 men; *M*_age_ = 22.28; *SD* = 3.45 years) participated in the experiment. Data cleaning resulted in a final data set of 254 participants (63 men; *M*_age_ = 22.40; *SD* = 3.52 years).

#### Materials

The materials were identical to the materials in Experiment 1a.

#### Procedure and design

The procedure and design were fully identical to Experiment 1a. The experiment took about 45–50 min.

### Results and discussion

#### Data cleaning

Mean accuracy was 83% versus 94% for the memory and lexical-decision task, respectively. We discarded data from five participants who performed the memory task below the threshold of 60 % (as in the previous experiments). We also deleted the data of participants who did not pass the 90% threshold in the lexical-decision task. The mean accuracy of the rest of the participants for the memory task was 84%, and for the lexical decision task, it was 95%. We ended up with a final set of 212 participants (50 men; *M*_age_ = 22.56; *SD* = 3.54 years).

As before, we next selected the trials in which participants had correctly responded to both tasks, leading to a data loss of 13.95% and 3.85%, respectively for the two tasks. As before, we discarded absolute outliers (3.34%) as well as relative outliers (1.87%). The means of the final set of RTs are shown in Table 3.

**Table 3:** Means and standard deviations per condition in Experiments 3–5

	Experiment 3		Experiment 4		Experiment 5	
Referent position	Dot position		Dot position		Dot position	
	Top	Bottom	Top	Bottom	Top	Bottom
Up	702(93)	694(88)	779(142)	770(138)	930(212)	943(210)
Down	715(91)	706(90)	769(133)	762(136)	991(222)	982(213)

#### Response-time analysis

We applied the same procedure as in previous experiments. The likelihood ratio test revealed a marginal main effect of the referent position: (χ2(1) = 2.87, *p =* .09, *d* = 0.39), showing faster RTs for “up” compared to “down” words. Also, the main effect of the dot position was significant: (χ2(1) = 12.18, *p<.*001, *d* = -0.06), demonstrating that people were faster to make decisions after memorizing the “bottom” dots than after memorizing the “top” dots. The model with interaction did not outperform the model without interaction (χ2(1) = 0.32, *p =*.57, *d* = -0.01). Planned comparisons revealed a main effect of dot position both in “up” words: (χ2(1) = 4.83, *p =* .028, *d* = -0.06), and in “down” words: (χ2(1) = 7.29, *p =* .001, *d* = -0.07) in the same direction.

As before, we performed an additional analysis, also taking into account trials in which participants had not correctly responded to the memory task. The interaction was not significant: (χ2(1) = 1.94, *p =*.164, *d* = -0.02). Planned comparisons revealed a main effect of dot position both in “up” words: (χ2(1) = 4.34, *p =* .037, *d* = -0.05) and in “down” words: (χ2(1) = 14.85, *p<.*001, *d* = -0.09), again in the same direction.

#### Accuracy analysis

No condition showed accuracy benefits over the others (all *ps >* .4). The percentage of correct responses per condition is shown in Table 4.

**Table 4:** The percentage of correct responses in Experiments 3–5

	Experiment 3		Experiment 4		Experiment 5	
Referent position	Dot position		Dot position		Dot position	
	Top	Bottom	Top	Bottom	Top	Bottom
Up	96%	96%	99%	99%	98%	97%
Down	95%	96%	95%	96%	98%	98%

### Discussion

Experiment 3 showed no evidence that keeping a location in working memory affects the decision about words with a spatial association. These results thus now pretty clearly suggest that the differences observed for the up-words in the analyses of Experiment 1a and Experiment 2 are not reliable and are not observed even in a highly powered experiment. We see at least two potential factors that may have contributed to not finding an effect in this and the previous experiments. First, lexical decision might be a too shallow task to see an influence of a location held in working memory on the processing of space-associated words. Second, the verticality of the working-memory task might not have been strong enough: Even if participants memorized a dot in the top versus bottom positions of the screen in different trials, they could always be sure that the test dot would appear at the same portion of the screen as the target dot (top or bottom). Thus, maybe verticality (up vs. down) was simply not salient enough in the present setup. Of course, both aspects – if turned out to be relevant – would point towards a more strategic, non-automatic activation of spatial attributes when processing space-associated words. However, as we are interested in the boundary conditions for when memorizing a location affects the processing of space-associated words, we decided to explore the relevancy of these factors in a further experiment. In the next experiment, we replaced the lexical-decision task (shallow) with a semantic task that taps into deeper processing. Also, we emphasized verticality by changing the non-matching dot positions to one in the opposite part of the vertical screen.

## Experiment 4

Experiment 4 was designed to explore further whether the position of a dot in working memory would affect decisions about a space-associated word if we “relax” the boundaries for the effect, namely by emphasizing verticality and by using a less shallow linguistic task. For the linguistic task we asked participants to decide whether the word referred to an occupation or not.

For the determination of sample size, we simulated a data set based on the results obtained in the study by Šetić and Domijan (2007), which involved a semantic task and observed a significant interaction between word meaning and presentation location. This study was the most similar study we found to the task employed in the current experiment. We then performed analyses of the simulated data and determined that we needed 100 participants to achieve 90% of the power for observing a potential interaction of the two factors. We thus aimed at collecting at least but ideally more than 100 participants for this experiment (again taking into account that effect sizes are often overestimated, see above).

### Method

#### Participants

Data were collected from 179 participants (41 men; *M*_age_ = 23.03; *SD* = 4.07 years). The cleaning procedure left us with 151 subjects (36 men; *M*_age_ = 22.78; *SD* = 3.28 years).

#### Materials

The stimuli were the 40 “up” and “down” words we used before. This set differed from the previous experiments only in terms of fillers: we have replaced the previous fillers with 80 nouns for the purpose of the task that referred to an occupation (e.g., *doctor*).

#### Procedure and design

We slightly modified the paradigm in an attempt to enhance the salience of the vertical dimension in the memory task. Specifically, in this experiment the second dot in the memory task, if mismatched with the first dot, appeared in the opposite part of the screen. We also replaced the lexical-decision task with a semantic task and changed the time of word presentation. In particular, we asked participants to remember the position of the dot and, when the word appeared at 3,000 ms, assess whether the word was related to an occupation or something else. The rest of the procedure was the same as in Experiment 3. The experiment took approximately 15–20 min. The design was the same as in Experiments 1–3.

### Results and discussion

#### Data cleaning

Mean accuracy was 95% and 97% for the memory and the semantic tasks, respectively. The memory task in Experiment 4 was easier than in the previous experiments because participants did not have to keep in mind the exact location of the dot but rather only its position in the upper versus lower part. We therefore changed the accuracy threshold to a minimum of 85% correct answers. The data of five participants had to be excluded because they did not meet this threshold. Two further participants did not pass the threshold of 90% of correct responses for the word task. The final set consisted of 147 participants (34 men; *M*_age_ = 22.8; *SD* = 3.2 years). The mean accuracy for these participants was 96% and 97% for the memory and word tasks, respectively.

As before, we based our RT analysis on trials in which participants had correctly responded to both tasks. This resulted in data loss of 6.16% and 2.6% for the memory and word tasks, respectively. We defined and omitted absolute (1.1%) and relative outliers (4.87%) as before. The means of the final set of RTs are shown in Table 3.

#### Response-time analysis

We applied the same analysis procedure as in Experiments 1–4. The base model consisted of fixed main effects for the referent position and dot position and random intercepts for participants and items. We compared the base model with the reduced models where one of the fixed effects was omitted. The likelihood ratio test revealed a main effect of the dot position (χ2(1) = 4.59, *p =* .032, *d* = -0.04) demonstrating that people were faster to make decisions about words after they saw a “bottom” dot than a “top” dot. The main effect of referent position was not significant (χ2(1) = 0.44, *p =* .506, *d* = -0.15). The further model comparison also showed that the model with interaction did not outperform the model without interaction (χ2(1) < 0.01, *p =* .971, *d* <0.01). Planned comparisons did not reveal any main effect of dot position, neither for “up” words: (χ2(1) = 2.36, *p =* .125, *d* = -0.04) nor for “down” words: (χ2(1) = 3.32, *p =* .127, *d* = -0.04).

Even if the current memory task was not as difficult as the previous one, we still decided to run the analysis in which we did not exclude trials in which participants had responded erroneously in the memory task. No interaction was observed: (χ2(1) < .001, *p =*.999, *d* < .001). Simple effects were not significant (all *ps >* .1).

#### Accuracy analysis

The accuracy analysis showed no effect (all *ps >* .1). The percentage of correct answers per condition is shown in Table 4.

### Discussion

In Experiment 4, we changed the task from a shallow task to a task requiring deeper semantic processing, and emphasized the concept of verticality in the memory task. However, even after this, the results of Experiment 4 speak against the hypothesis that the rehearsing of a position in memory influences the decision about words with spatial associations. We are therefore pretty confident in concluding that keeping a location in spatial working memory does not influence the processing of spatially associated words, contrary to what would have been expected based on embodied accounts of language processing. We think that this conclusion is justified based on the fact that we went through a lot of efforts in four experiments to produce conditions where this effect – if reliable – should have been observed. However, we also think that these repeated null results would be even more convincing in the light of an experiment that actually does show an effect similar to the one under investigation. This was the aim of Experiment 5.

As mentioned above, in one of their studies, Pecher et al. (2010) found an effect of word position on the processing of space-associated words when the task was explicitly space-related, namely when participants had to decide whether the word’s referent is typically found in the sky (“up”) or in the ocean (“down”). To further delineate the boundary conditions of our hypothesized effect of memorizing a location in working memory on the processing of space-associated words, we decided to run one final experiment. In this experiment we did the following. First, we excluded fillers, because previous research showed that the presence of filler words without any space association (which we had in our previous experiments) may reduce spatial effects in an experiment (Dudschig & Kaup, 2017; for a similar effect with color words, see Tsaregorodtseva et al., 2023). Second, we made the linguistic task space-related by directly asking the participants about the spatial association of the words, pressing the “O” key for an up-word (“oben” in German), and the “U” key for a down-word (“unten” in German).

## Experiment 5

Experiment 5 aimed to determine whether the effect of memorized position would affect the activation of the spatial component of semantics in spatially related words when the participants were engaged in a direct spatial task. The experiment was preregistered (https://aspredicted.org/ev3k5.pdf).

### Method

#### Participants

Since we also use a semantic task in Experiment 5, we kept the target sample size of participants the same as in Experiment 4. As in Experiment 4, we aimed to collect at least 100 participants, ideally more. As a result, we collected data from 154 participants (28 men; *M*_age_ = 22.59; *SD* = 4.53 years). The cleaning procedure led us to a data set of 137 participants (24 men; *M*_age_ = 22.16; *SD* = 3.98 years).

#### Materials

We used the stimulus set we used before, namely, the 40 “up” and 40”down” words without extra fillers.

#### Procedure and design

In Experiment 5, we changed the response configuration and the linguistic task. Participants were asked to decide whether the word referred to objects they can see when they raise their heads or to objects they can see when they lower their heads. We asked them to press “O” on the keyboard in the former case and “U” in the latter case. The time of word presentation was limited to 3,500 ms. The rest of the procedure was the same as in Experiment 4.

### Results and discussion

#### Data cleaning

The mean accuracy was 95% and 96% for the memory task and the linguistic task, respectively. We discarded the data of seven participants who either did not pass the threshold of 85% for the memory task (N = 4) or 90% for the linguistic task (N = 3). The mean accuracy after elimination was 96% and 97%, respectively. The final set of participants comprised 130 people (22 men; *M*_age_ = 22.09; *SD* = 3.97 years).

**Fig. 2:**
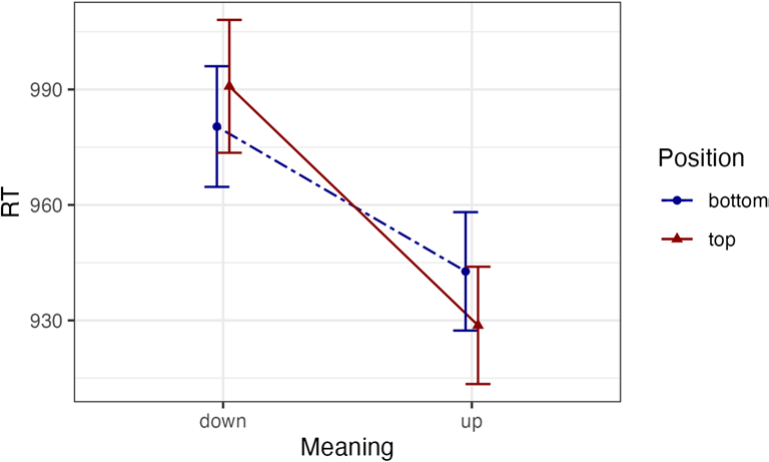
Response times in the semantic task of Experiment 5. Error bars denote 95% within-subjects confidence intervals calculated as recommended by Morey (2008)

As before, we only included trials, in which the participants had correctly responded to both tasks. By this we lost 3.92% and 2.26% of the data for the memory and the word task, respectively. According to our criteria there were no absolute outliers, and eliminating the relative outliers resulted in a loss of 5.36% of the data. The means of the final set of RTs are depicted in Fig. 2 (see also Table 3).

#### Response-time analysis

The data were analyzed in the same way as in Experiments 1–4. The analysis showed no main effect of the dot position (χ2(1) = 0.14, *p =* .711, *d* = 0.01), but a main effect of referent position (χ2(1) = 9.85, *p =* .002, *d* = 0.73), showing that people were faster making decisions about “up” compared to “down” words. More importantly for our present purposes, the model with interaction significantly outperformed the model without interaction (χ2(1) = 4.7, *p =* .03, *d* = -0.06).

Planned comparisons revealed a marginal main effect of dot position for “up” words: (χ2(1) = 3.64, *p =* .056, *d* = 0.06), where participants showed faster RTs in congruent compared to incongruent positions (930 ms vs. 943 ms, respectively). Numerically, there was also an advantage for congruent compared to incongruent conditions for “down” words (982 ms vs. 991 ms, respectively), which, however, was not significant: (χ2(1) = 1.36, *p =* .244, *d* = 0.06).

As before, we ran an additional analysis in which we did not exclude the erroneous trials from the memory task. This analysis showed the same pattern of results. Namely, we did not observe a main effect of the dot position (χ2(1) = 0.09, *p =* .761, *d* = 0.01), but observed a main effect of referent position (χ2(1) = 9.64, *p =* .002, *d* = 0.73). The interaction was significant (χ2(1) =5.55, *p =* .018, *d* = -0.05). Planned comparisons revealed the main effect of dot position in “up” words (χ2(1) = 3.93, *p =* .047, *d* = 0.06) with the means being 931 versus 945 ms, for top and bottom positions, respectively. The effect was not observed in the “down” words (χ2(1) = 2.01, *p =* .157, *d* = 0.06) with the means being 994 versus 982 ms for top and bottom positions.

#### Accuracy analysis

The accuracy analysis revealed no difference between conditions (all *ps >* .2). The percentage of correct responses per condition is shown in Table 4.

### Discussion

In comparison with Experiment 4, in Experiment 5, we replaced the indirect semantic task with the direct semantic task, where we asked participants about the spatial associations of the words. We thus made the spatial associations of the words directly task relevant. We additionally intensified the activation of the vertical dichotomy by assigning the “O” and “U” keys associated with *oben* and *unten* (“above” and “below”) as responses. As a result, we now observed an effect of the remembered position in working memory on the processing of space-associated words. In particular, actively maintaining a position in working memory facilitates the processing of a word associated with that position when the task is to decide about the spatial associations of the word.

Overall, the results of Experiments 1–5 revealed that if the sensorimotor experience is preactivated, it enhances the activation of the congruent location during language comprehension; however, the effect is highly task- and context-dependent. In particular, we observed the small (*d* = -0.06) effect of remembered position on spatially related words only when verticality was drastically salient. In our view, the observed effect in Experiment 5 provides further convincing evidence that the null-results obtained in the previous four experiments targeting more automatic influences of remembered location onto language processing were true null results and not the result of a faulty paradigm or poorly conducted studies. In our view, the results of our five experiments thus clearly show that processes in visual-spatial working memory do not facilitate or hinder the regular processing of words with spatial associations. The results of our experiments thus did not produce the much-needed evidence for strong versions of the embodied-cognition framework where linguistic processes are directly influenced by compatible or incompatible processes in the non-linguistic domain. Nevertheless, we conducted additional analyses to further corroborate our conclusions.

## Post hoc analysis

We first pooled all the data together and analyzed it with the factors of *compatibility* (referent position and memorized position: compatible vs non-compatible) and *experiment.* The results confirmed the result of the individual experiments: there was a significant interaction between compatibility and experiment (χ2(5) =12.55, *p =* .028), showing that the compatibility effect indeed differed between experiments. Next, we ran Bayes factor analyses for the individual experiments, to gain more information about the validity of our interpretation in terms of a null-effect in Experiments 1 through 4.

### Bayes factor and Bayesian analysis

As we did not observe an interaction in our experiments except in Experiment 5, we ran a Bayes factor analysis using the Bayes factor package to test how substantial the absence (Experiments 1–4) or presence of the effect (Experiment 5) really was. The results of this analysis corroborated our conclusions by showing that in most of the experiments, the evidence against the interaction ranged from moderate to very strong (Experiments 1–4), and in Experiment 5 where we actually observed an interaction, the evidence in favor of this interaction was only anecdotal (see Table 5).

**Table 5:** The Bayes factor for the interaction in Experiments 1–6

Courses of analysis	1	1а	2	3	4	5
1	0.09609149 ±3.07%	0.04337901 ±6.74%	0.0429115 ±3.92%	0.03063826 ±2.81%	0.03038869 ±4.69%	0.3288159 ±2.68%*
2	0.09040499 ±2.91%	0.03795987 ±2.18%	0.1337446 ±3.17%	0.07036941 ±3.97%	0.0311313 ±2.94%	0.5467043 ±12.33%*

Asterisks denote a significant interaction in experiments

Given the fact that the random effect structure in our models was simple because of the convergency issues in LMEM, we also ran a Bayesian analysis, using the *brms* package with a more complex random structure. We fitted Bayesian hierarchical linear models to RT as a function of dummy-coded factors of referent position and dot position and their two-way interaction. The models included random intercepts for items and participants and the interaction between the referent position and dot position as random slopes for participants. The Bayesian analysis confirmed our interpretations. The results showed low probability (less than 95%) of an interaction in the first four experiments and 95% probability for an interaction effect in Experiment 5. We present a brief summary of the Bayesian analyses in the online folder together with the materials of the current studies.

## General discussion

It has been shown that actively maintaining a location in visual-spatial working memory recruits similar resources as processing stimuli at a particular location in perception, namely attention and eye-movement activity (Awh et al., 1998; Ferreira et al., 2008; Theeuwes et al., 2009). At the same time, compatibility effects in language comprehension suggest that processing words referring to referents with a typical location in space also direct attention to a corresponding location in space and prepares corresponding eye-movement activity (Dudschig et al., 2012; Dudschig et al., 2013; Ostarek et al., 2018). Embodied theories of language comprehension explain this latter result by assuming that words activate experiential traces stemming from prior experience with the respective referents, which are integral to meaning (Barsalou, 1999). We conducted five experiments combining the two lines of research. More specifically, we asked whether maintaining a location in working memory would or would not affect the subsequent processing of words with a spatial association. We assumed that word processing might be facilitated in the case where the location held in memory is compatible with the typical location of the word’s referent and hindered in case the two locations are incompatible.

In all of our experiments, participants were first shown a visual stimulus at a particular location and participants were asked to keep the location (or shape) in memory so that they would later on be able to judge whether a second visual stimulus appeared at the same or a different location (had the same or a different shape). In between the presentation of those two visual stimuli, participants were presented with a linguistic task, responding to a word referring to an entity that is typically encountered in the upper or lower part of the visual field (e.g., *root* = down; *roof* = up) possibly intermixed with other linguistic stimuli. In the first three experiments, the linguistic task was lexical-decision and therefore required rather shallow linguistic processing. In Experiment 4, we presented participants with a deeper semantic task which however was still unrelated to the spatial associations of the words in question. Finally, in Experiment 5, we explicitly asked participants to judge whether the words referred to something typically found in the upper versus lower part of the visual world. Thus, whereas the spatial associations of the words were task irrelevant for the first four experiments, they provided the imperative stimulus in the last experiment and were therefore task relevant in this experiment. The results of our experiments were rather clear. We observed an interaction of stimulus position and word meaning (indicative of an effect of maintained location in memory on word processing) only in the last experiment, in which the words were explicitly processed with respect to their spatial associations. In other words, no evidence could be obtained that regular word processing is indeed influenced by prior compatible or incompatible processing in the non-linguistic domain, and this was the case when the linguistic task was rather shallow (lexical decision) but also when it required deeper processing targeting the meaning of the words (occupation-task). The fact that the relevant interaction was observed in the last experiment, in which participants explicitly judged the spatial attributes of the words shows that any alternative explanation in terms of the unsuitability of the paradigm are unsustainable.

It could be argued that it is not fully clear whether the task is the decisive factor. After all, we did not only change the linguistic task in Experiment 5, making spatial associations task relevant and using response keys with a spatial meaning (using “U” for “unten” [down] and “O” for “oben” [up]). In addition, compared to all of the other experiments we conducted, Experiment 5 was the only one in which there were no linguistic stimuli without a spatial association. Thus, although we took out the regular fillers (words requiring a “yes” response but not having a spatial association) in Experiment 4, we still had 80 words referring to an occupation (required for our semantic task in this experiment). We think that the presence or absence of fillers probably doesn’t have a large effect when the task already draws attention to the relevant meaning dimension. After all the usual explanation why the absence of fillers lets some effects emerge is that the relevant dimension becomes more salient without fillers. In any case, this specific question although important in principle doesn’t seem central to our point here. If an influence of spatial properties of non-linguistic processing on linguistic processing can only be observed when space is made highly salient by task or experimental context, then this shows that regular word processing is not influenced by non-linguistic activity, be it because the task was not space-related or too many words were without a space relation.

A commonality between Experiment 4 and 5 concerns the non-matching dot positions. In both experiments, we emphasized verticality by having non-matching dot positions in the opposite part of the vertical screen. We think that this might have prompted participants to activate the concepts of “up” and “down,” possibly using a verbal code for memorizing the dot location. Consequently, the compatibility advantage observed in Experiment 5 could be attributed to semantic priming between the preactivated concept (up vs. down) and the required response to the word (up vs. down). Importantly, the absence of an interaction in Experiment 4 suggests that activating a verbal code in itself does not seem sufficient for observing a compatibility effect. This is in opposition to traditional priming effects, where priming occurs even if the response to the target does not require a decision about the relevant dimension (Heyman et al., 2015). From this perspective, the results of Experiment 5 can probably be best interpreted within the framework of conflict effects, such as the Simon effect (Khalid & Ansorge, 2013; Luo & Proctor, 2021), where an irrelevant stimulus dimension influences responses on the relevant response dimension, leading to a stimulus-response (S-R) compatibility effect. In Experiment 5, the physical dot position that is to be retained in memory is irrelevant for the task but overlaps with the required response to the words (“up” or “down”). Thus, the spatial code activated by the dot’s location influences the spatial response, resulting in the compatibility effect observed in Experiment 5. Accordingly, the absence of an interaction in Experiment 4 can be attributed to the lack of an overlapping response dimension, even if participants were required to retain the spatial position in memory. We believe that future research, perhaps involving the suppression of potential verbal activity, could further illuminate the nature of the effect observed in Experiment 5. Nevertheless, our series of experiments clearly demonstrates that the interaction between the retained dot position in memory and the spatial attributes of word meanings occurs only under very limited conditions.

It is worth noting that, in contrast to Awh et al. (1998) study, whose paradigm we adapted for our research, we did not track eye movements. However, we believe that this matter does not significantly impact our study. Our reasoning is the following. In the study by Awh et al. (1998), researchers investigated whether a memorized location would influence the recognition of a figure (choice stimulus) that had a specific place on the screen. In their case, monitoring participants’ eye behavior was crucial to ensure that participants indeed returned their eye-gaze from the location to be memorized to the center of the screen. This was important because it made sure that participants actually retained the location in their memory rather than simply shifting their eyes from the location to be memorized to the location of the choice stimulus. In contrast, our study examined whether a memorized location would influence the decision about a word stimulus, which, as we hypothesized, should trigger the simulation of a spatial location (e.g., the word sun evoking an “up” simulation). In our study, participants needed to return from the location to be memorized (dot processing) to the center of the screen where the word stimulus was placed, in order to make a decision on the word. The task setup thus guaranteed that participants would return their eyes to the center of the screen, superseding the necessity to control participants” eye-movements.

One might of course wonder if participants could have used their peripheral vision to recognize the word, potentially enhancing their performance in the memory task. However, we do not consider this likely for several reasons. First, using peripheral vision would have been quite challenging given the considerable distance between the dot and the word in many trials. Additionally, the duration of word presentation was limited. Finally, participants were “punished” with a 5,000-ms frozen screen in case of an incorrect response in the lexical decision task. It was therefore more advantageous for participants to focus on solving the lexical decision task as quickly and accurately as possible and this would require directing their eye-gaze to the center of the screen where the word appeared. In fact, the relatively high accuracy rates for the lexical decision task in our experiments suggest that participants indeed focused on the word in the center of the screen rather than maintaining their gaze on the memorized dot during the retention interval. Therefore, we are certain that the lack of eye behavior monitoring should not be regarded as the explanation for the absence of effect of memorized dots on the activation of spatial semantics in linguistic stimuli.

So, where does this leave us? We set out with the hope that we could observe an effect of a memorized location on the processing of spatially related words which would allow us to argue that sensorimotor representations are *functional* for language understanding as assumed by embodied views of language comprehension. However, the results of the five experiments conducted in this study suggest that this is not the case, at least not as far as spatial meaning dimensions are concerned. Instead, the results suggest that the effect of memorized location on spatially related words only occurs under very specific conditions, namely when the experimental context and task directs attention to vertical space. These findings are in line with the results of other studies looking at spatial meaning dimensions, such as Pecher et al. (2010), who found the respective compatibility effects only when employing a spatially-related task, and with Shaki and Fischer (2023), who obtained an interaction between the implicit spatial meaning of a word and the position of a subsequent probe only when the task required access to the spatial component of semantics. In addition, Berndt et al. (2018) showed that a particular visual background highlighting spatial attributes was necessary for an effect of word position on anagram solving to be observed (see above).

Overall, our results thus seem more in line with hybrid accounts of language understanding (e.g., Dove, 2009; Louwerse & Jeuniaux, 2010), assuming that modal meaning representations, such as re-activated experiential traces, are just one of the many meaning representations that humans have at their disposal. According to these accounts, experiential simulations are only used under certain conditions and are not required to understand language. Our results are therefore also in line with the increasing evidence that human cognition in general involves different sorts of mental representations, differing in the degree to which they are more modal or more amodal (Kaup et al., 2022).

## Data Availability

All data generated during this research can be found online (https://osf.io/p76ug/). Experiments 3 (https://aspredicted.org/gf7mw.pdf) and 5 (https://aspredicted.org/ev3k5.pdf) were preregistered.

## Footnotes

1 Considerations of sample size for our initial experiments were based on a study by Lachmair et al. (2011; Experiment 1) that seemed most comparable to our planned study with respect to task and stimuli. In this study, the same set of stimuli was used with a lexical decision task, examining the interaction between upward/downward movements and the spatial meaning of words. We first simulated a data set based on the parameters provided by the Lachmair et al. (2011) study and then ran a linear mixed-effects model (LMEM) analysis 1,000 times for each chosen number of participants (20, 40, 60, 100). From these simulations, we determined that in order to achieve a statistical power of at least 90% for observing a potential interaction between our two factors, we would need a sample size of at least 40 participants. This number also was similar to the sample size used in other studies investigating the interaction between upward/downward movements or cue position and word meaning (Dudschig & Kaup, 2017; Lachmair et al., 2011; Tsaregorodtseva & Miklashevsky, 2015). Considering that our study was to be conducted online and involved a more complex dual-task procedure that might lead to more noise in the data, we decided to double sample size and collect at least 80 participants in our initial experiments.

2 We used overlapping response keys in the two tasks to minimize effort for the participants by avoiding the need to memorize two separate sets of keys. This approach aligns with other studies in the literature where researchers utilized similar paradigms with shared keys for multiple tasks (e.g., Awh et al., 1998; Belopolsky & Theeuwes, 2009). It should also be noted that our main focus in this paper was on the participants’ first task. Potential interference effects through overlapping response keys would probably be more an issue for RTs in the participants’ second task which was not in the focus of our analyses.

3 This outlier criterion might seem rather strict. However, it is important to note that we decided to use this outlier criterion before conducting our experiments and applied it consistently throughout all experiments. Importantly, analyses with a number of different outlier cutoffs, namely 3 SDs, 3.5 SDs, and 1.5 SDs all yielded the same qualitative outcomes as the analysis with our initially chosen cutoff.
